# Aachen-Heerlen annotated steel microstructure dataset

**DOI:** 10.1038/s41597-021-00926-7

**Published:** 2021-05-26

**Authors:** Deniz Iren, Marc Ackermann, Julian Gorfer, Gaurav Pujar, Sebastian Wesselmecking, Ulrich Krupp, Stefano Bromuri

**Affiliations:** 1 Center for Actionable Research of Open Universiteit, Valkenburgerweg 177, 6419 AT Heerlen, The Netherlands; 2 Steel Institute, RWTH Aachen University, Intzestr. 1, 52072 Aachen, Germany

**Keywords:** Mechanical properties, Computer science

## Abstract

Studying steel microstructures yields important insights regarding its mechanical characteristics. Within steel, microstructures transform based on a multitude of factors including chemical composition, transformation temperatures, and cooling rates. Martensite-austenite (MA) islands in bainitic steel appear as blocky structures with abstract shapes that are difficult to identify and differentiate from other types of microstructures. In this regard, material science may benefit from machine learning models that are able to automatically and accurately detect these structures. However, the training process of the state-of-the-art machine learning models requires a large amount of high-quality data. In this dataset, we provide 1.705 scanning electron microscopy images along with a set of 8.909 expert-annotated polygons to describe the geometry of the MA islands that appear on the images. We envision that this dataset will be useful for material scientists to explore the relationship between the morphology of bainitic steel and mechanical characteristics. Moreover, computer vision researchers and practitioners may use this data for training state-of-the-art object segmentation models for abstract geometries such as MA islands.

## Abstract (summary)

**Table:** 

Measurement(s)	area of martensite-austenite in steel • Polygon
Technology Type(s)	scanning electron microscopy • Annotation
Factor Type(s)	steel manufacturing process parameters

Machine-accessible metadata file describing the reported data: 10.6084/m9.figshare.14045789

## Background & Summary

Bainitic steels have many uses in civil engineering such as production of rails, pipelines, and other forgings, as well as in the automotive industry to manufacture steering knuckles or chassis components. The versatile use is related to its outstanding combination of high strength and toughness^1–3^. These properties are depending on chemical composition, transformation temperature, and cooling rate as they influence the bainitic microstructure.

The microstructure is composed of a carbon-depleted primary phase and a carbon-rich-secondary phase^4^. Bainite arises from a mixture of shear mechanism without diffusion and a diffusion-controlled formation, but it remains unsettled which mechanism is dominant^5^. The ratio of diffusion-less and diffusion-controlled parts causes differences in the sub-structures. This led several researchers to introduce different classification schemes. The most prominent classes are lower and upper bainite^6^. Depending on temperature, upper bainite typically transforms mostly diffusion-controlled at higher temperatures. Lower bainite transforms mainly diffusion-less at lower temperatures^7^. These classes can be visually identified by carbide formation within the secondary phase (upper bainite) or in the primary phase (lower bainite)^8^. Varieties increased with advances in the steel production (higher precision in the alloying of steels) and with precisely controlled heat treatments. Besides carbides, Zajac *et al*.^9^ incorporates incomplete transformation products in the secondary phase and classifies these microstructures as “degenerate bainite”. Martensite-austenite (MA) islands are an example of degenerate constituents. While MA islands in lower and upper bainite occur as elongated up to film-like structures (high length:width ratio), a slow continuous cooling can lead to the formation of granular bainite containing block-type (length:width ≈1) MA islands^10^. Further classifications are described in^11^. Those classifications can be used to predict the mechanical behavior, where the existence of coarse MA islands is an indicator for a decrease of impact energy^12,13^. Unfortunately, prior research lacks quantitative measurements with meaningful numbers of MA islands. Often only the average size was used to interpret this sub-structure in bainite. Given the manifold morphologies of MA, the average size appears insufficient to describe the correlation between microstructure and mechanical properties.

Material scientists have been working on the identification, explanation, and replication of those structures. Understanding the mechanisms that lead to the formation of those specific morphologies is necessary to describe their impact on the mechanical properties. However, different sizes, shapes, and structures in bainite cause, until now, a manifold interpretation based on a subjective feature description by the expert who currently examines the microscopic images manually. Correlative metallography is a common approach to retrieve complementary knowledge about sub-structures^14,15^. Correlative means to couple different experiments to gain morphological, crystallographic, or chemical data of the same spatial region in the sample. Otherwise, some of the microstructural information remains hidden^16^. Thus, the characterization of bainite microstructures on microscopy images poses a challenge, and detecting a clear correlation between microstructure and mechanical properties is extremely difficult. To improve reliability and reproducibility, recent developments in machine learning provide a promising approach to tackle the challenge of microstructure description by computer vision.

Two challenges in machine learning that are relevant to the MA detection task are *object detection* (i.e., detecting the existence of the object and specifying the location of the object on the image) and *object segmentation* (i.e., drawing the boundaries around the object that separates it from the rest of the image). Object detection applications have seen an improvement in performance in the last years, thanks to the advent of new deep learning methodologies and architectures. Deep learning allowed researchers to move from a sliding window approach to generate bounding boxes to new strategies for specifying regions of interest. Several examples are Regions with Convolutional Neural Network features (RCNN)^17^ and You Only Look Once (YOLO)^18^, which generate regions with respect to a fixed grid by regressing the position of the bounding boxes. Similar to YOLO, Single Shot Multi-Box Detector (SSD) produces the prediction of the bounding boxes by means of a single forward pass on top of a convolutional neural network network^19,20^. Although, the architectural developments show promise, training object detection and segmentation models require a significant amount of high-quality annotated data, and expert-annotated image sets are rare to come across.

The automated identification of microstructures in steel poses a specifically intriguing challenge in machine learning. Steel micro-imagery consists of very complex images having a significantly large amount of abstract patterns appearing on the image in comparison to the application of object detection or segmentation in other problem domains such as pedestrian and vehicle detection^21,22^. Therefore, the expected benefit of transfer learning (i.e., applying the knowledge gained while training to solve a particular problem to a different yet similar problem) is limited.

With this dataset descriptor, we contribute to both materials science and machine learning fields by providing (a) an image collection of steel microscopy on which blocky-type morphologies in bainite microstructures that are annotated by experts, (b) metadata regarding the morphology of these structures. Materials scientists may use the dataset to conduct bainite-related research, and machine learning practitioners may utilize the data to train and test object detection and segmentation models.

## Methods

**Fig. 1:**
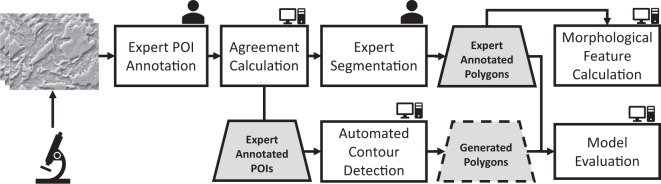
The steps of the data generation process. The images of various steel samples were acquired via a scanning electron microscope. The MA islands that appear on these images were annotated redundantly by multiple experts as points-of-interest (POI). The set of POIs was refined into a smaller set based on the agreement of the experts using the spatial proximity of the individual annotations. Subsequently, the experts drew polygons on the boundary of the MA islands that were previously marked by the majority of experts. The agreed-upon POIs were also used to guide the automated contour detection around the MA islands. The generated polygons were benchmarked against the expert-annotated polygons. Finally, the morphological characteristics of expert annotated polygons were calculated.

In this section, we describe our method for generating the data, and measuring and calculating features. Fig. 1 represents the steps of our research process that includes data collection, annotation, feature calculation, and evaluation.

### Scanning electron microscopy

**Fig. 2:**
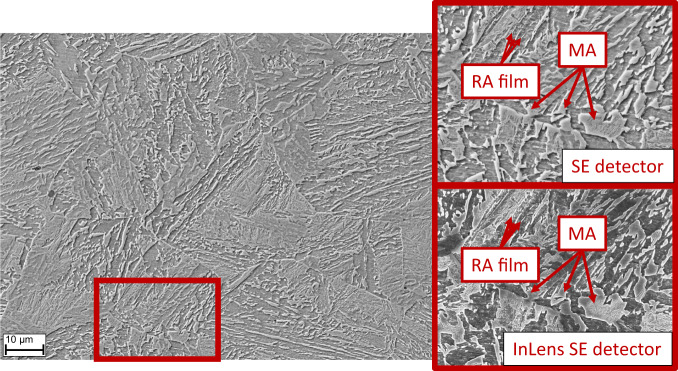
SEM micrographs of a bainitic microstructure taken by two different detectors. The marked MA islands are composed of martensite in the center and austenite located at the outer rim causing a topography effect^29^. In contrast, retained austenite films reveal another topography effect in this type of microstructure.

In the literature on bainitic microstructures, the characterization appears often ambiguous^9,23–26^. Nevertheless, no widely accepted quantitative characterization is available on steels containing bainite in the microstructure. This makes it difficult to draw conclusions from microscopic constituents to the macroscopic mechanical properties. Bainite consists of a carbon-depleted primary phase and a carbon-rich secondary phase with variations of morphologies depending on heat treatment and alloying elements^7^. The secondary phase can form so-called MA islands^27^. The MA structure becomes critical if carbon from adjacent phases has not enough time during cooling to penetrate the entire island. The resulting carbon depletion causes instability in the centre of the island resulting in fresh martensite transformation, while the outer rim remains only partly austenitic^28^. Fig. 2 shows an example of a bainitic microstructure with MA islands originated from two different microscope detectors. MA constituents occur in different shapes with highly elongated or rather round shaped structures. The indicated MA islands and the retained austenite films show both a topographic effect in the image causing sometimes difficulties to separate elongated MA from austenite films.

The martensite fraction in these islands cause a local hardness gradient and act therefore as brittle spots prone to produce microcracks^29^. Large MA islands commonly inherit large martensitic areas, but in the case of elongated islands, carbon can diffuse and stabilize more austenite in the MA islands during cooling.

Bainitic microstructures can consist of constituents with the order of magnitude on the sub-micron or even nanometer scale that cannot be imaged by light optical microscopy due to the limited spatial resolution. Scanning electron microscopy (SEM) provides high resolution images (resolution limit less than 10 nm) of a probed surface suitable for refined and more complex microstructures. Here, emitted electrons from a field emission gun are focused on the sample surface. These primary electrons are scattered on the surface. Inelastic scattering excites electrons located close to the surface. These excited electrons cause emission of secondary electrons. Backscattered electrons and secondary electrons can be detected revealing local chemical (Back-Scattered Electron Detector) or topological (Scanning Electron Detector) gradients, respectively.

Electron backscatter diffraction (EBSD) is a powerful instrument to gather morphological (grain size, area, and shape) and crystallographic (misorientation and texture) data of the microstructure. Together with atom probe tomography (APT), EBSD maps can be overlapped with element mappings to identify chemical gradients in MA constituents^28^. Unfortunately, EBSD is a time expensive method which requires also additional time for postprocessing. Therefore, EBSD (or APT) is not suitable for an analysis on larger surface areas or on a larger set of samples. For this reason, SEM images were chosen to generate efficiently a high quantity of image data suitable to be used as training input for computer vision models.

Prior to the SEM analysis, samples were metallographically prepared by grinding and polishing with a surface finish of 1 µm. Nital etchant (3% nitric acid) was used to reveal the bainitic microstructure for SEM. The austenite fraction inside MA islands is mechanically unstable. Thus, mechanical loading (grinding and polishing) during sample preparation can cause a transformation of meta-stable austenite. This effect needs be minimized before interpreting microstructural data to account only for the material-related occurrences. Prolonged polishing with oxide polishing suspensions or (electrolytic) etching provides a deformation-free surface, so austenite stays intact inside the MA islands^30^. Only secondary electron detectors were used that yield topographic information to take micrographs of local spots covering an area of nearly 650 µm^2^. In case of the studied MA structures, the islands show a topographic effect in the SEM image. This effect originates from the etching response and manifests in a brighter appearance from the outer rim to the centre of the island. For larger islands, etching even reveals refined lath structures in the centre of the islands. The complexity of the given task lies in differentiating the MA islands from occasional carbides (bright points) and film-type austenite. These film-type structures occur as elongated and bright films with a thickness in the range of nanometer. The images were acquired in a Zeiss Sigma field emission gun SEM with secondary electron and Inlens detectors by Oxford. In this procedure, SmartSEM software was used. The SEM was operated at 15 kV with a working distance below 10 mm and with 60 µm aperture.

### Metadata of the steel samples

In total, 10 steel samples with different chemical and heat treatments were investigated. The characteristics of the steel samples are associated with each image and provided in the metadata table. These characteristics include a label that indicates the chemical composition of the samples, phase transformation temperature, the direction of the photography axis, the distance of the sample to the edge, magnification level, and the tilt angle. The details of these characteristics are provided in the data records section. Prior to the annotation step, the experts were trained to identify MA islands based on a thorough microstructural characterization of bainite microstructures from preselected samples^31^.

### Bainite annotations

#### Annotation platform

We used a web-based 2D image annotation platform for the acquisition of spatial annotations on image sets. Essentially, the annotation platform consists of two parts: a control panel that shows the task description, and the buttons to submit the annotations, and a canvas object that displays a single image from the dataset with a transparent overlay that records the spatial coordinates of annotators’ mouse clicks. The software displays images one by one, in a random order. Two separate modes of operation are supported by the annotation software that were used in different steps of this study. Depending on the mode of operation, users are allowed to either mark multiple POIs or draw one polygon through consecutive clicks.

#### POI annotation protocol

In total, 2.580 images were annotated separately and redundantly by three domain experts. At this step, the annotation task consisted of marking a point that is inside the blocky MA region. The annotators were instructed to provide one and only one POI marker per structure. Therefore, we make the assumption that every marker put by a particular annotator belongs to exactly one MA structure that is visible on the image. However, markers that are placed by different annotators may indicate the same MA structure which may mean that the experts agree on the existence of a particular structure. Images were displayed in 1024 × 768 resolution, and the coordinates of annotated POIs were recorded in that resolution scale. For completeness, the annotators marked all MA structures that they could identify on the images.

**Fig. 3:**
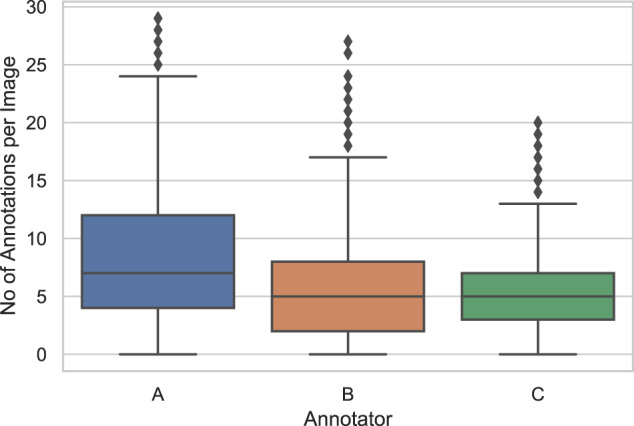
The distribution of the number of POIs marked on images by the experts A, B, and C.

The annotators were also able to flag an image to be removed due to low quality features such as artefacts from sample preparation, lens focus-shift, etc. Images that have been flagged by any of the annotators were removed from the dataset, leaving a sum of 1.768 images. The descriptive statistics of the number of annotations collected by the annotators are provided in Table 1 and Fig. 3.

**Table 1:** The descriptive statistics of POIs marked by experts A, B, and C. N denotes the number.

	number of images	number of POIs	mean	standard deviation	min	max
A	1.768	14.982	8,47	5,46	0	29
B	1.768	9.670	5,47	4,14	0	27
C	1.768	9.785	5,54	3,23	0	20

#### POI agreement

**Fig. 4:**
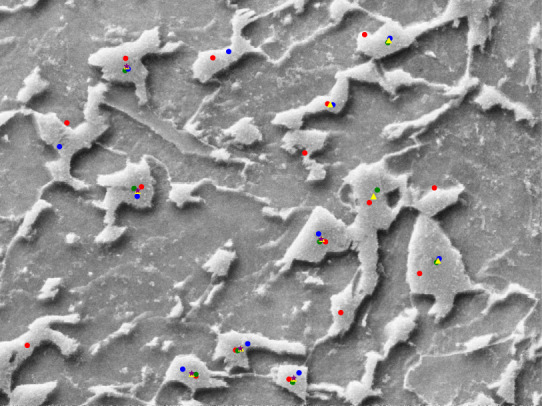
Example visualization of POIs marked by the expert annotators. Red, green, and blue colored points are the POIs provided by annotators. Yellow points mark a POI that was agreed by two annotators. Purple colored points denote the agreement of three annotators.

Even for the experts, detecting bainite structures is a challenging task. As an attempt to decrease the number of falsely identified bainite structures, we used majority decision which is a commonly applied quality assurance method in information retrieval^32^. The idea behind the majority decision is that if multiple annotators agree on one annotation separately, the likelihood of an error is decreased. For this spatial annotation task, we used Euclidean Distance measurement along with a threshold to calculate agreement. Thus, if the distance between the markers placed by different annotators is smaller than the threshold, the markers are assumed to indicate the same point of interest. This threshold value differs based on the characteristics of the images in terms of object frequency and distribution. The experts have decided to set the threshold as 0.4 micrometers (~30 pixels under 4000x magnification level), by manually analyzing the POIs marked. An example image with individual annotations and agreed-upon POIs can be seen in Fig. 4. In order to predict the agreement on multiple points, we made a pairwise Euclidean Distance measurement of all POIs marked by the annotators. If two or three POIs (each marked by a different annotator) were closer to each other in comparison to the threshold value (0.4 micrometer), we accepted the centroid of those POIs as the agreed POIs.

The number of POIs resulted by the agreement of all three annotators was 2.913, while the number of POIs agreed by two or three annotators was 8.909. At this step, we removed 63 images from the dataset because there were no agreed-upon POIs marked on them.

#### Polygon annotation (Expert Segmentation) protocol

**Fig. 5:**
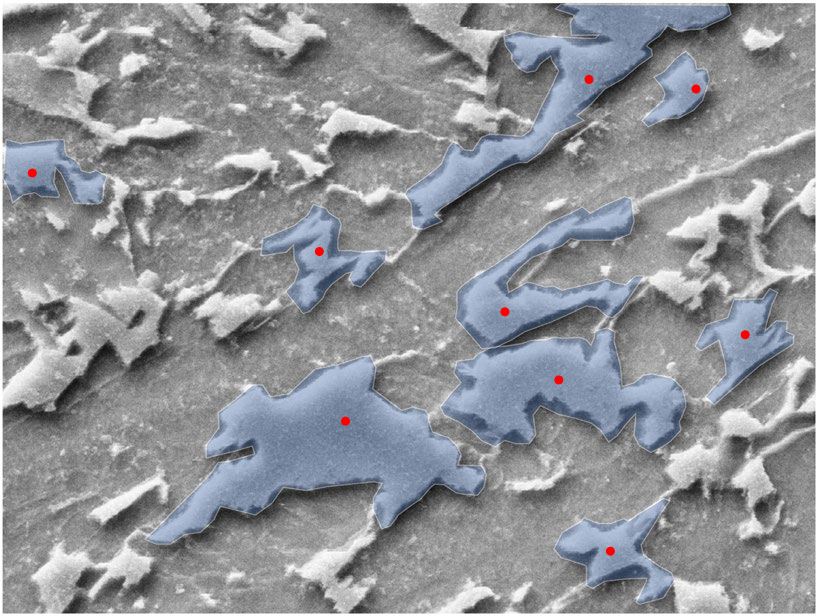
Segments that are drawn by an expert. The red dots mark the agreed-upon POIs that were collected in the POI annotation step.

After having the list of agreed POI coordinates, the expert annotators performed the segmentation task using the annotation software. Each of the 8.909 agreed POIs were displayed on the base image and the annotator was asked to draw a bounding polygon around the blocky structure. In order to avoid distraction, the expert was provided with one POI at a time and drew only one polygon to segment a single bainite structure at a time. After the completion of each segmentation task, the annotation software displayed a randomly chosen POI from the agreed-upon POI list. The polygons were represented as an ordered list of point tuples with x and y coordinates that resulted from the mouse clicks of the expert annotators. An example visualization of such segments is shown in Fig. 5.

### Morphological feature calculation

Morphology of the MA constituents is studied in the literature, often to find correlations with the mechanical properties of the steels. The coarsening of these structures is shown to cause deterioration of the impact energy^12^. Aside from the size of the structures, the shape of MA islands is also studied in the literature to identify the factors that affect the morphology of these structures^33^. The analysis of both size and shape of microstructure depend on manual measurement using optical imagery or EBSD data that may potentially lead to incomplete and inconsistent results. The data generated for this study covers in total 10 steel samples, exposed to different heat treatments. Therefore, the images reflect microstructures with both, a different processing history (effect of temperature and cooling) and different chemical composition (effect of alloying elements). Beyond the size of the MA islands as a critical factor, further morphological data can be extracted to find correlations with mechanical properties of certain steels. In case of non-equiaxed islands, different shapes can be addressed by obtaining an aspect ratio (ratio of height and width). For instance, a microstructure with round shaped MA constituents and another with an elongated morphology will have a different shape, but the same area. Thus, the average size of MA may not be sufficient to describe the resulting difference in mechanical properties for both cases. Therefore, the area, perimeter, aspect ratio, and compactness^34^ are more accurate measures to identify critical MA islands. Polygon compactness measure ranges between 0 and 1. 1 indicates maximal compactness (i.e., a perfect circle) and 0 indicates complete lack of compactness. Formulas (1) and (2) show how aspect ratio and polygon compactness were calculated where *P* denotes a polygon.

1 $$aspect\_ratio(P)=\frac{height(bounding\_box(P))}{width(bounding\_box(P))}$$

2 $$compactness(P)=\frac{4\times \pi \times area(P)}{perimeter{(P)}^{2}}$$

## Data Records

All data tables and images that are explained in this section are accessible at Figshare^35^.

### Image set

The image data set includes 2.580 TIFF files and their PNG equivalents for displaying on web browsers for annotation purposes^35^. TIFF files are the original outputs of the SEM and PNG equivalents are created to be displayed on web browsers for annotation purposes. Note that only 1.705 of the images have polygon annotations associated with them. The remaining image files are excluded due to suboptimal image quality or there are no agreed-upon MA-islands indicated on them. These images are provided in a separate folder.

### Steel sample metadata

The image metadata table shows the image file name, steel-sample composition type, sample-preparation temperature, direction, distance to the edge, magnification level, and angle of the SED^35^. The description of the fields in the table are listed below.

- **Type** describes the chemical composition of the steel. The specifics of the composition are not included in this dataset. Type property is only provided as a classification feature to indicate compositionally different classes.
- **Temperature** is the temperature of phase transformation. The possible values are 400 °C and 500 °C.
- **Direction** indicates the direction on which axis the photograph has been taken. It can be ‘Horizontal’ for left-to-right and ‘Vertical’ for top-to-bottom.
- **Distance** is the distance to the edge of the sample cross-section in either horizontal or vertical direction. The maximum distance in horizontal direction can be 24 mm and in vertical direction 13 mm. These distances are a result of the deformation of the sample by compression.
- **Magnification** is the magnification level while taking images with the electron microscope. All images in this dataset were recorded under 4000x magnification.
- **Angle** describes the tilt-angle (that was created by selecting a specific scanning electron detector) that was used during the photo making procedure. ‘tilt’ indicates a tilt angle to create a bigger shadow on the sample. This helps to see the different topographic elevations of structures better. ‘perpendicular’, means that the electron ray flows perpendicular to the camera lens which is also called in-lens. By using a perpendicular electron ray, the image usually gets brighter than otherwise.

### POI annotations

POI annotations table shows the entire set of annotations provided by all three experts. Each entry consists of the image name and an X, Y coordinate tuple that represents the offset coordinates of the point that was marked to indicate an MA structure by one of the experts. This table also includes the coordinates of MA structures that were agreed by either two or three experts.

- **Image_url**: The name of the base image.
- **A**: The list of POI annotations provided by the expert A.
- **B**: The list of POI annotations provided by the expert B.
- **C**: The list of POI annotations provided by the expert C.
- **doubleAgreement**: The list of POI annotations on which at least two experts agreed.
- **tripleAgreement**: The list of POI annotations on which three experts agreed.

The POI lists are stored as coordinate-tuples. An example list that contains four points is as the following. [(616, 319), (820, 286), (986, 522), (880, 521)]

### Polygon annotations (Expert Segmentation)

Each entry in the expert segmentation table represents a polygon that marks the boundary around an MA structure. The POIs that lead to the drawing of a particular polygon annotation is also provided within the data table. Polygons are defined by a series of consecutive point tuples such as the following example. [(720, 288), (725, 294), (731, 300), (737, 30), …]

- **Image_url**: The name of the base image.
- **point**: The point that marks the MA structure. The point is defined with one tuple of coordinates.
- **polygon**: The polygon that marks the boundary of the annotated MA structure.

### Morphological features

The morphological features data table contains the measured and calculated features of the MA islands that are represented by polygon objects. The table contains the image_url, point, and polygon fields of the expert segmentation table and enriches them with the morphological features that are described below. The points and polygons are represented in Shapely geometry objects with the purpose of facilitating measurements and calculations.

- **Image_url**: The name of the base image.
- **point_shapely**: The Shapely representation of the point. This point resides within the polygon of the annotated MA island.
- **poly_shapely**: The Shapely representation of the polygon that represents an MA island.
- **polygon_area**: The area of polygon that represents an MA island in pixel-units.
- **polygon_area_metric**: The area of the polygon that represents an MA island in micrometers.
- **polygon_perimeter**: The perimeter of the polygon that represents an MA island in pixel-units.
- **polygon_perimeter_metric**: The perimeter of the polygon that represents an MA island in micrometers.
- **height**: The height of the rectangle that bounds the polygon in pixel-units.
- **width**: The width of the rectangle that bounds the polygon in pixel-units.
- **aspect_ratio**: The height of the bounding box of the polygon divided by the width of the bounding box of the polygon. This is an indicator of the shape of an MA island that is represented by the polygon.
- **polygon_compactness**: Calculated Polsby-Popper compactness^34^.
- **rotation_angle_polygon**: The angle of tilted minimum bounding rectangle^36^.

### Outcome of a baseline contour detection model

This table contains polygons (contours) that are automatically created by the baseline model. The purpose of this table is to provide a benchmarking opportunity for computer vision researchers and practitioners. These polygons were created with a simple contour detection method that uses optimal parameters for the set of images that were used in this study.

- **Image_url**: The name of the base image.
- **point**: The expert-annotated point that marks the MA structure.
- **polygon**: The expert-annotated polygon that marks the boundary of the MA structure.
- **point_shapely**: The representation of ‘point’ in Shapely data format.
- **poly_shapely**: The representation of ‘polygon’ in Shapely data format.
- **contour_polygon_shapely**: The representation of the automatically detected contour in Shapely data format.

### Evaluation

The evaluation table consists of three additional features on top of the baseline contour detection model; area of the expert-annotated polygons, area of the automatically detected contours, and the Intersection-over-Union (IOU) as a measure of segmentation accuracy.

- **area_contour**: The area of the automatically detected contour in pixel-units.
- **area_poly**: The area of the expert-annotated polygon in pixel-units.
- **IOU**: The area of intersection of the expert-annotated polygon and the automatically detected contour divided by the area of the union of them. This is a measure of segmentation accuracy.

## Technical Validation

We ensured the technical validation of the dataset and our curation approach focusing on three areas; POI annotations, polygon annotations, and the usefulness of the dataset for training and evaluating object segmentation models.

MA islands appear as abstract geometries and complex structures on microscopy images. This makes it difficult even for expert material scientists to identify these structures correctly, completely, and consistently. To ensure the accurate annotation of MA structures, we used a redundancy-based quality assurance approach, in which we selected the annotations based on the majority decision of multiple experts^32^. Out of 14.982, 9.670, and 9.785 individual annotations, we were able to identify 8.909 MA islands that were separately annotated by at least two experts, and 2.913 that were agreed-upon by three experts.

Following the polygon annotation phase, the quality of polygon annotations was controlled by two activities. First, we automatically checked if the polygons are valid. The validity of a polygon means that the polygon rings are closed, edges do not intersect with each other, and the edges do not overlap. Secondly, the outcome polygons were individually displayed on base images and carefully visually inspected by the curators.

Lastly, we used contour detection to segment the already-detected microstructures. More specifically, the contouring procedure uses POIs to automate the consecutive step, which is to draw the boundaries of the MA islands that contain the POIs indicated by experts. The contouring procedure detects the boundaries based on stark changes in color and intensity. Therefore, it produces polygons that are much more complex and detailed than expert-annotated polygons. The purpose of applying the contouring procedure in our study is threefold. First, we aimed at seeing whether there is a pattern that could be captured to train models. We assume that the change of color and intensity comprises useful information regarding the boundaries of MA islands. The comparison of contouring and expert-annotated polygons allows us to evaluate the importance of color and intensity changes in detecting boundaries of MA islands. Second, we aimed at evaluating contouring as a supporting tool for the polygon annotation task. Due to the complexity and abstractness of the MA islands -and potentially many other microstructures that appear in steel-, contour detection methods can assist the annotator by fine-tuning the curvature of the polygon boundary that is indicated by POIs placed by the annotator. An example of a similar usage is the magic wand tool that is available in many image processing software products. Finally, contour detection provides a baseline model for segmentation. Therefore, any machine learning model trained for the segmentation of MA islands must overperform the baseline model in terms of accuracy.

Subsequently, we compared the outcome of the baseline contour detection method against the expert-annotated polygons. The baseline method yielded 0,35 IOU. This may suggest that the change in color and intensity are a part of the decision taken by the experts when drawing the boundaries of MA islands. Even though contour detection may not replace a machine learning segmentation model, it has the potential to ease the polygon annotation task and improve the quality of expert-annotations. Further research is required to improve the annotation procedure as well as to train segmentation models that can detect MA island boundaries accurately. To that end, this dataset comprises a rich source for training such machine learning models.

## Usage Notes

We envision two usage scenarios for this dataset. Firstly, material scientists can use the data to explore the relationship between a variety of characteristics of steel with the MA islands that exist in them. Secondly, machine learning researchers and practitioners can use the annotated shapes as high-quality training data to train state-of-the-art object segmentation models^37^. The methods to visualize the polygons and points on the images are also provided within the code to allow researchers to visually inspect the geometries on the detected MA islands.

## Data Availability

All codes that were used in the preparation and the analysis of this dataset are made available along with the dataset and in our code repository^38^. Codes are written in Python language. Specifically, two Jupyter Notebooks were provided; one that contains the code that describes the data tables and calculates the morphological feature, and another for contour detection and evaluation of the segmentation model. The code includes all custom methods, references to common libraries, and a full set of stepwise instructions to replicate the calculations in this study.
